# Protective effects of new aryl sulfone derivatives against radiation-induced hematopoietic injury

**DOI:** 10.1093/jrr/rraa009

**Published:** 2020-03-16

**Authors:** Jian Cao, Hongyan Li, Renbin Yuan, Yinping Dong, Jing Wu, Meifang Wang, Deguan Li, Hongqi Tian, Hui Dong

**Affiliations:** Tianjin Key Laboratory of Radiation Medicine and Molecular Nuclear Medicine, Institute of Radiation Medicine, Chinese Academy of Medical Science & Peking Union Medical College, Tianjin 300192, China

**Keywords:** aryl sulfone derivatives, hematopoietic cells, radioprotection

## Abstract

The hematopoietic system is sensitive to radiation. In this research, new aryl sulfone derivatives (XH-201 and XH-202) containing a nitrogen heterocycle were designed and synthesized and their radio-protective efficacies with regard to the hematopoietic system were evaluated. XH-201 administration significantly increased the survival rate of mice after 8.0 Gy total body irradiation (TBI). The results showed that XH-201 treatment not only increased the white blood cells, platelets counts and the percentage of hematopoietic progenitor cells and hematopoietic stem cells in mice exposed to 4.0 Gy TBI but also decreased DNA damage, as determined by flow cytometric analysis of histone H2AX phosphorylation. In addition, our data demonstrated that XH-201 decreased the mitochondrial reactive oxygen species (ROS) levels in hematopoietic cells. Overall, these data suggest that XH-201 is beneficial for the protection of the hemoatopoietic system against radiation-induced injuries.

## INTRODUCTION

Ionizing radiation (IR) exposure may take place in radiological accidents or terror attacks. Exposure to IR can damage multiple organ systems, including the gastrointestinal tract, the cerebral vasculature and the hematopoietic system [1], but conventional radiation therapy remains important for cancer treatment. Therefore, it is important to protect radiosensitive tissues and organs from IR-induced damage. At present IR is an undeniable danger and can lead to catastrophic consequences for health [2, 3]. Endeavors aimed at developing medically effective radiation countermeasures were initiated more than 60 years ago. Nonetheless the number of drugs that can be used in clinical practice is limited. Therefore, it is critical to develop medical preparedness and countermeasures for radioprotection.

**Scheme 1.**
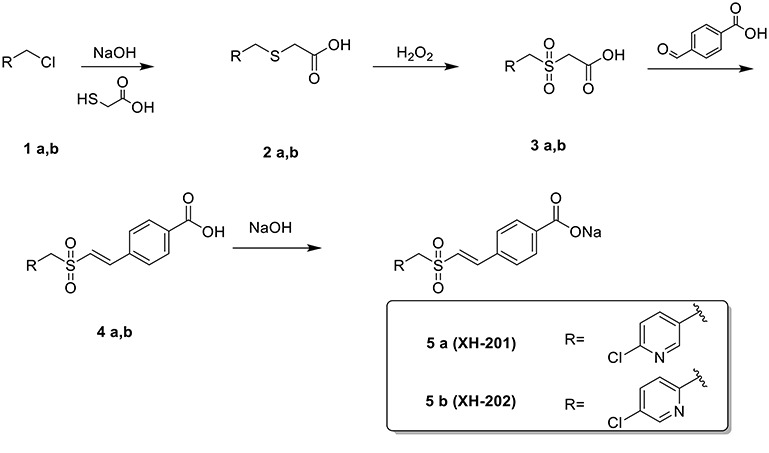
Preparation of XH-201 and XH-202

Despite a range of scientific advances related to radioprotectors, currently there is no drug approved by the Food and Drug Administration (FDA) for acute radiation syndrome (ARS). Amifostine (WR2721) is the only radioprotector approved by FDA for mitigating the side-effects of radiotherapy [4–6]. But WR2721 itself has serious side-effects, e.g. vomiting, nausea and hypotension [7, 8]. Recently, many radiation countermeasures such as 5-androstenediol, PrC-210, CBLB502, ON01210 and BIO300 have been investigated [2]. Ex-Rad (ON 01210.Na) is a chlorobenzyl sulfone derivative developed by Onconova Therapeutics (Newtown, PA, USA) as a radioprotector for modifying cell cycle distribution patterns in cancer cells subjected to radiation therapy [9, 10]. Furthermore, a range of novel aryl sulfone derivatives have been prepared recently whose stereochemical and electronic structures were explored, aiming to obtain more effective radioprotective agents [11,12]. Nitrogen heterocycles are the most basic structural characteristics of drug molecules [9]. So, we think it is necessary to modify heterocycles containing nitrogen, based on an aryl sulfone structure, and explore the relationship between structure and activity. By modifying the physicochemical properties of a nitrogen-containing heterocyclic compound, XH-201 and XH-202 were designed and prepared rationally (Scheme 1) and the radioprotective effects of the resulting compounds were evaluated *in vitro*.

The hematopoietic system is very sensitive to ionizing radiation [13–16]. IR-induced acute bone marrow (BM) suppression leads to ARS resulting in infection, bleeding, anemia and other clinical manifestations. Exposure to moderate to high doses of total body irradiation (TBI) causes life-threatening damage known as BM suppression [17, 18]. Apoptosis occurs in hematopoietic progenitor cells (HPCs) and a small number of hematopoietic stem cells (HSCs) after exposure to IR, resulting in acute BM suppression in a short period of time [19]. The hematopoietic system is widely used for evaluating the radioprotective effects of new compounds.

In the present study, firstly, we investigated the protective effects of XH-201 and XH-202 *in vitro*, then we investigated the protective effects of XH-201 on TBI-induced hematopoietic injury and the underlying mechanisms involved in these effects by using our well-established and well-characterized mouse model.

## MATERIALS AND METHODS

### Procedure for the preparation of XH-201 and XH-202

First mercaptoacetic acid (5.3 g, 57 mmol) was added to aqueous NaOH (2.3 mol/L, 50 mL) slowly at 25°C. Then compound **1a** (9.0 g, 56 mmol) (see Scheme 1) was added. The reaction mixture was stirred at 50°C for 3 h. The progress of the reaction was monitored by TLC. The mixture was cooled with ice-water, and adjusted to pH 3 with aqueous HCl (10 mol/L). The mixture was stirred for 1 h at 0°C. After that it was filtered, the filtered cake was washed with ice-water and dried to give compound **2a** (6.1 g) as a yellow solid. ^1^H NMR (400 MHz, DMSO-d6)) δ 8.34 (s, 1H), 7.83 (d, J = 8.2 Hz, 1H), 7.62–7.34 (m, 1H), 3.85 (s, 2H), 3.17 (s, 2H). ^13^C NMR (100 MHz, DMSO-d6)) δ 171.60, 150.33, 149.28, 140.63, 134.09, 124.57, 33.22, 32.10. ESI-MS: m/z = 218 (M + H) ^+^; HR-MS (ESI): m/z [M + H] ^+^ calcd for C_8_H_9_ClNO_2_S: 218.0043, found: 218.0062.

To a solution of compound **2a** (5.0 g, 23 mmol) in HOAc (25 mL), aqueous H_2_O_2_ (13 g, 30%) was added slowly for 15 min. Then it was warmed to 50°C and stirred for 9 h. The progress of the reaction was monitored by TLC. Then the mixture was cooled and added to ice-water (100 mL). It was then left to stand overnight and filtered. The filtered cake was dried to give compound **3a** (1.6 g) as a white solid. ^1^H NMR (400 MHz, DMSO-d6)) δ 8.40 (d, J = 2.2 Hz, 1H), 7.88 (dd, J = 8.3, 2.5 Hz, 1H), 7.60 (d, J = 8.2 Hz, 1H), 4.74 (s, 2H), 4.28 (s, 2H).^13^C NMR (100 MHz, DMSO-d6)) δ 164.89, 152.21, 151.04, 142.66, 124.82, 124.32, 57.35, 55.69.ESI-MS: m/z = 250 (M + H)^+^; HR-MS (ESI): m/z [M + H]^+^ calcd for C_8_H_9_ClNO_4_S: 249.9941, found: 249.9959.

Under a nitrogen atmosphere, compound **3a** (1.6 g, 6.6 mmol), 4-formylbenzoic acid (1.1 g, 7.2 mmol) and benzylamine (0.25 mL) were added to 10 mL of HOAc. Then the reaction mixture was heated to reflux. The progress of the reaction was monitored by TLC. The mixture was cooled and filtered and the filtered cake was washed with cooled EtOH. Then the crude compound **4a** was dried and added to aqueous NaOH (10 mL, 0.6 mol/L). The mixture was heated to reflux for 15 min. It was then cooled to 25°C and stirred for 2 h. The suspension was filtered to give XH-201 (1.1 g) as a white solid. ^1^H NMR (400 MHz, D_2_O) δ 8.27 (s, 1H), 7.79 (d, J = 7.8 Hz, 3H), 7.52 (d, J = 7.9 Hz, 2H), 7.48–7.04 (m, 3H), 7.04–6.83 (m, 1H), 4.58 (s, 2H). ^13^C NMR (100 MHz, D_2_O) δ 174.58, 151.26, 150.95, 146.99, 142.46, 139.37, 133.90, 129.39, 128.75, 124.91, 123.68, 123.36. ESI-MS: m/z = 360 (M + H) ^+^; HR-MS (ESI): m/z [M + H] ^+^calcd for C_15_H_12_ClNNaO_4_S: 360.0073, found: 360.0052.

The preparation procedure of XH-202 was similar to XH-201 (for characterization of XH-202 see online supplementary material). The purities of XH-201 and XH-202 were >98% (detected by HPLC), as shown in Supplementary Figs S9 and S10, see online supplementary material.

### Biological evaluation

#### Reagents

A formulation of XH-201 and XH-202 was used in these studies. The following antibodies were purchased from eBioscience (San Diego, CA, USA): anti-mouse Ly-6A/EA (Sca-1)-PE, rat IgG2a- PE (Isotype Ctrl), CD117 (c-kit)-APC, rat IgG2b-APC (Isotype Ctrl), biotin-conjugated CD5, CD4, CD8, CD45R/B220, Ly6G/Gr-1, CD11b, Ter-119 and PerCP-conjugated streptavidin antibodies [20]. RPMI 1640 medium was purchased from Gibco (Grand Island, NY, USA). Melatonin was purchased from Tixiai Chemical Industry Co., Ltd (Shanghai, China). BD Cytofifix/Cytoperm buffer was purchased from BD Biosciences (San Diego, CA, USA). MitoSOX red mitochondrial superoxide indicator was obtained from Life Technologies (Grand Island, NY, USA). Rabbit anti-γH2AX antibody was obtained from Cell Signaling Technology (Danvers, MA, USA) and FITC-conjugated goat anti-rabbit antibodies from Abcam (Cambridge, MA, USA).

#### Cell culture and irradiation

BM cells flushed from mouse femurs with PBS were adjusted to 1 × 10^6^ cells/ml with RPMI 1640 (Roswell Park Memorial Institute-1640) and plated into 96-well plates. BM cells were cultured aseptically in RPMI-1640 supplemented with 10% (v/v) fetal bovine serum (FBS) and penicillin (100 units per/mL)/streptomycin (100 g/L), pH 7.2, in a 5% CO_2_ humidified atmosphere at 37°C before irradiation. The plates were irradiated with the needed dose, 1.0 Gy, for the cell survival assay at a dose rate of 1.0 Gy/min. Cells irradiation was done at the X-radiation facility of the Institute of Radiation Medicine, Chinese Academy of Medical Science and Peking Union Medical College, Tianjin, China.

#### 1,1-Diphenyl-2-picrylhydrazyl radical scavenging assay

Eight different concentrations of XH-201 and XH-202 were prepared in DMSO (from 25 μM to 1000 μM) and mixed with 200 μM 1,1-diphenyl-2-picrylhydrazyl (DPPH) in ethanol. In addition, eight different concentrations of melatonin were prepared in water (from 25 to 1000 μM) and also mixed with 200 μM DPPH in ethanol as a control. Then, the mixtures of samples were left to stand at room temperature in the dark for 30 min. At the endpoint of the reaction, the mixtures were measured at 517 nm using a spectrophotometer microplate reader. Absolute ethanol or water was used as a reagent blank to calibrate the instrument. Parallel operations were performed four times to take the average value. The percentage of inhibition was calculated by the equation [21]

$$ \%\kern0.5em \mathrm{Inhibition}=\left[\left({\mathrm{A}}_{\mathrm{control}}\hbox{-} {\mathrm{A}}_{\mathrm{sample}}\right)/{\mathrm{A}}_{\mathrm{control}}\right]\times 100 $$

where A_control_ is the absorbance of the control and ${\mathrm{A}}_{\mathrm{sample}}$ is the absorbance of the test sample

#### Cell viability assays

The BM cell viability assay was conducted as in our previous studies [22, 23]. In brief, BM cells (1 × 10^6^ cells/ml) were plated into 96-well plates. The compounds XH-201 and XH-202 were dissolved in DMSO to a concentration of 0.02 mol/L and diluted in culture medium to the concentrations needed. The cultured cells were treated with the synthesized compounds (10^−4^, 10^−5^, 10^−6^, 10^−7^, 10^−8^ mol/L). After 1 h of incubation, the cells were exposed to 1.0 Gy of X-irradiation. Cell survival after 18 h of irradiation was measured by the luminescence-based CellTiter Glo TM assay (Promega, Madison, WI, USA) according to the manufacturer’s recommended protocols [24]. The luminescence of each well was read using a multimode microplate reader (Infinite M200, TECAN, Switzerland).

#### Animals

Male C57BL/6-Ly-5.1 (Ly5.1) mice weighing 20–22 g were purchased from Beijing HFK Bioscience Co, Ltd. (Beijing, China). The mice were bred at a certified animal care facility at the Institute of Radiation Medicine of Peking Union Medical College (IRM-PUMC); all mice were used at ~8–10 weeks of age and had weights of 20–22 g. All mice were randomly divided into different groups 1 week prior to the study to allow for acclimatization. All of the animal experiments in our study were approved by the Institutional Animal Care and Use Committee of IRM (No.2019009).

#### TBI irradiation and XH-201 administration

Male Ly5.1 mice used in the survival experiments were randomly assigned into the following five groups (*n* = 10): control, TBI, TBI + low-dose XH-201 (250 mg/kg), TBI + middle-dose XH-201 (500 mg/kg) and TBI + high-dose XH-201 (750 mg/kg). The TBI + XH-201 groups had XH-201 administered subcutaneously twice at 24 h and 30 min before irradiation. Mice were exposed to a lethal dose (8.0 Gy, for the survival study only) of TBI using an Exposure Instrument Cammacell-40 ^137^Cs-irradiator (Atomic Energy of Canada Ltd) at a dose rate of 0.88 Gy/min.

For the remaining experiments, mice were divided into the following three groups (*n* = 5): control, TBI (4.0 Gy) and TBI (4.0 Gy) + XH-201. Individual mice in the TBI + XH-201 group were administered a dose of 500 mg/kg XH-201 by subcutaneous injection 24 h and 1 h before a sub-lethal dose (4.0 Gy) of TBI. Control mice were sham-irradiated and received injections of vehicle. The mice were treated as described above and were killed 10 days after exposure to irradiation.

#### Peripheral blood cell and BM mononuclear cell counts

For the analysis of blood counts, peripheral blood from the orbital sinus was collected using a micropipette coated with an anticoagulant and analysed on a Celltac E hematology analyzer (Nihon kohden, Japan) and expressed as ×10^9^ cells/femur. The following measurements were recorded: white blood cell (WBC), red blood cell (RBC), hemoglobin (HGB), hematocrit (HCT) and platelet (PLT) counts. BM mononuclear cells (BMMNCs) were flushed from mouse femurs with PBS after the mice were euthanized and were counted and expressed as × 10^6^ cells/femur [25,26].

#### Detection of hematopoietic progenitor cells and hematopoietic stem cells by flow cytometric analysis

In brief, BM cells were stained with biotin-conjugated lineage antibodies for surface markers before being isolated by flushing both tibias and femurs with sterile phosphate-buffered saline (PBS). The cells were filtered and counted prior to staining with antibodies. The frequencies of hematopoietic progenitor cells (HPCs) (Lin^−^Sca1^−^c-kit^+^) and hematopoietic stem cells (HSCs) (Lin^−^Sca1^+^c-kit^+^) were detected using a BD Accuri C6 Flow Cytometer (BD Bioscience, San Jose, CA, USA) [27]. The data were calculated and analysed by BD Accuri C6 software.

#### Analysis of the levels of BM ROS via flow cytometry

For the detection of ROS, 5 × 10^6^ BM cells were incubated with biotin-conjugated antibodies specific for murine CD5, CD11b, CD45R/B220, Ter-119 and Gr-1 and then stained with streptavidin-PerCP, anti-Sca-1-PE and anti-c-kit-APC. Then the cells were incubated with MitoSOX (5.0 μM)) for 20 min at 37°C. The levels of ROS in HSCs were analysed by measuring the mean fluorescence intensity (MFI) of oxidized MitoSOX [28]. For each sample, a minimum of 100 000 Lin-cells were acquired [29]. The data were analysed using the BD Accuri C6 software.

#### Analysis of γ-H2AX phosphorylation

For the analysis of γ-H2AX, BM cells were processed as described for the experiments with BMMNCs, HPCs and HSCs, fixed and permeabilized with a cytofix/cytoperm buffer (BD Biosciences) before being stained with anti-γ-H2AX antibody according to the manufacturers’ instructions. H2AX phosphorylation in BMMNCs, HSCs and HPCs was analysed on the basis of the MFI of γ-H2AX staining in BMMNCs, HSCs and HPCs as assessed by flow cytometry. Data acquisition was performed on a BD Accuri C6 (BD Bioscience, San Jose, CA, USA) and analysed using the BD Accuri C6 software [29].

### Statistical analysis

All of these data were analysed using GraphPad Prism 6 software with Welch’s t-test (significantly different was defined as *P* < 0.05). The differences between treated and control groups were examined by using one-way ANOVA, LSD *post hoc* test and are presented as average ± SEM. Survival analyses were performed using the log-rank test.

## RESULTS

### Chemistry

We first synthesized compound **2** by using mercaptoacetic acid as a nucleophilic reagent, then the sulfides (**2**) were oxidized to obtain the corresponding sulfones (**3**) with aqueous H_2_O_2_ in acetic acid. Compounds **4** were synthesized from the reaction of **3** with 4-formylbenzoic acid. Finally, XH-201 and XH-202 were obtained from compound **4** by forming a sodium salt. Their structures were characterized by ^1^H NMR, ^13^C NMR and HR-MS spectra analyses.

### Biological evaluation

#### Determination of antioxidant activity

**Fig. 1.**
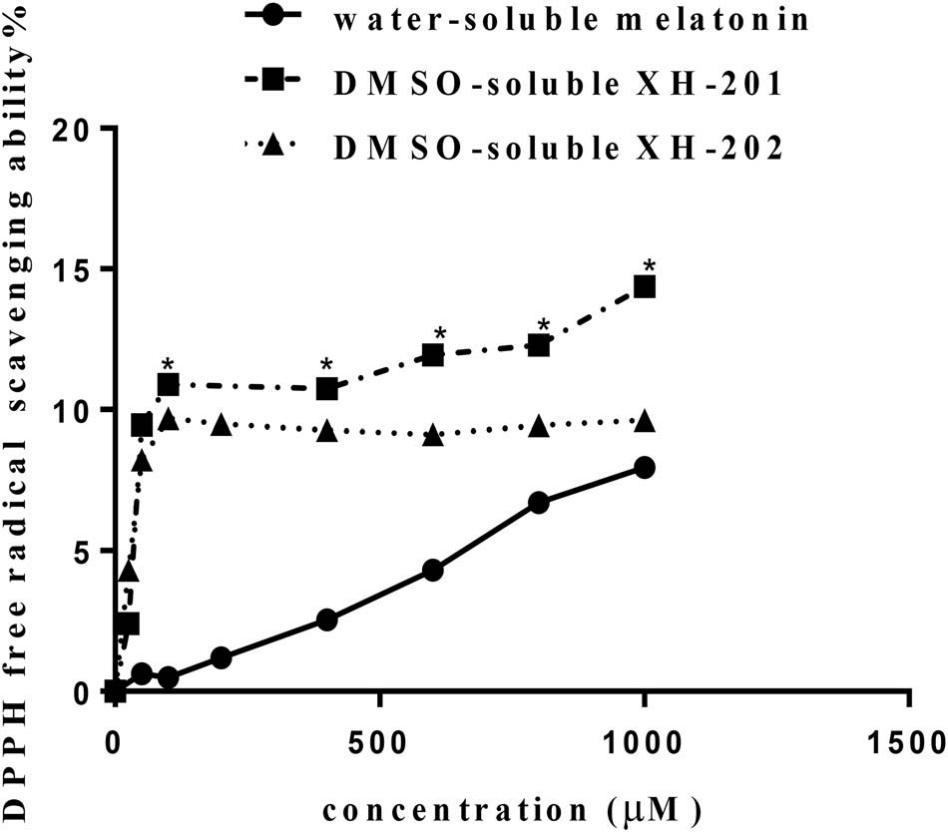
Effects on DPPH free radical scavenging ability of different compounds (XH-201, XH-202 and melatonin). Eight different concentrations of XH-201, XH-202 and melatonin (from 25 μM to 1000 μM) were detected by DPPH at 517 nm. ^*^Significant difference vs melatonin (*P* < 0.05).

In accordance with antioxidant capacity, XH-201 and XH-202 were evaluated by the DPPH radical scavenging assay, as shown in Fig. 1, and the concentration of the compounds was found to have a direct positive relationship with the scavenging rate. Compared with melatonin, the scavenging rate of XH-201 reached a significant level at 1000 μM. Moreover, at 100 μM, XH-201 and XH-202 showed relatively high scavenging rates; the rate tends to be stable afterwards, which indicated that XH-201 and XH-202 had a good effect on scavenging of hydroxyl radicals. The results suggested that XH-201 has more potential ability to scavenge hydroxyl radicals than XH-202.

**Fig. 2.**
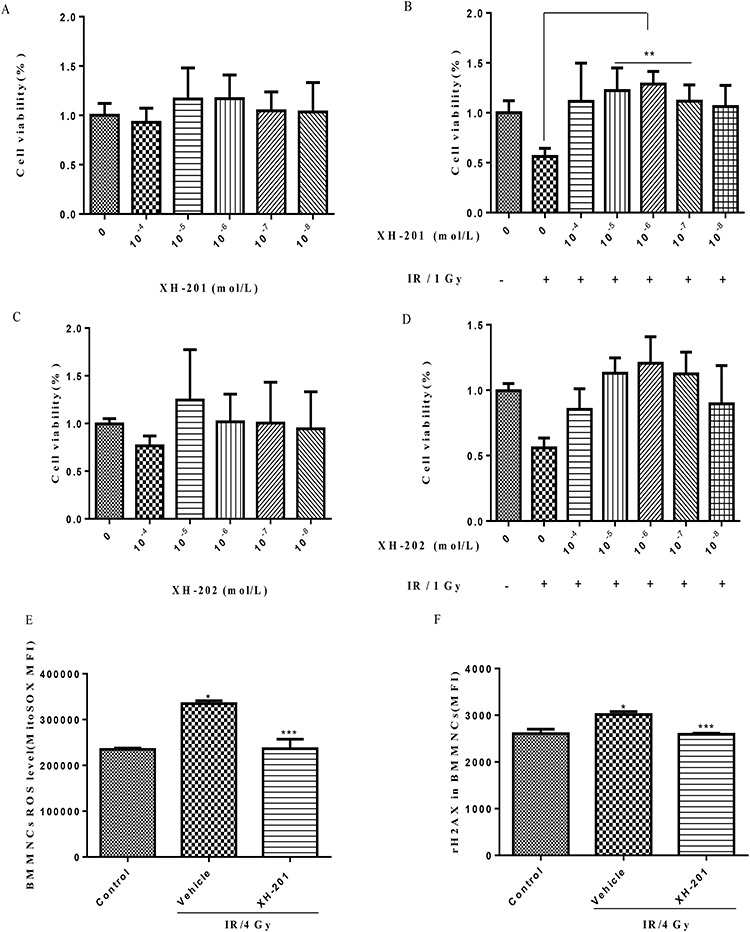
Evaluation of the radioprotective activity and cytotoxicity of XH-201 and XH-202 *in vitro*. The cells were sham-irradiated as a control or irradiated with 1.0 Gy after receiving different concentrations of XH-201 and cultured for 18 h. Cell viability was monitored using the luminescence assay, as described in the text. The other part cells were sham-irradiated as a control or irradiated with 4.0 Gy after receiving 10^−6^ mol/L XH-201 and after 30 min we examined the ROS levels of BMMNCs and histone H2AX phosphorylation among three groups. (**A**) Cell toxicity of XH-201; (**B**) viability of cells treated with 1.0 Gy irradiation after adding the required concentration of XH-201; (**C**) cell toxicity assay of XH-202; (**D**) cells treated with 1.0 Gy irradiation after adding the required concentration of XH-202. (**E**) ROS levels in BMMNCs treated with 4.0 Gy irradiation *in vitro*; (**F**) H2AX phosphorylation in BMMNCs treated with 4.0 Gy irradiation *in vitro*. The data were analysed by unpaired *t-*test. ^*^P < 0.05 vs control, ^**^P < 0.05 vs 1.0 Gy, ^***^P < 0.05 vs 4.0 Gy, *n* = 5.

**Fig. 3.**
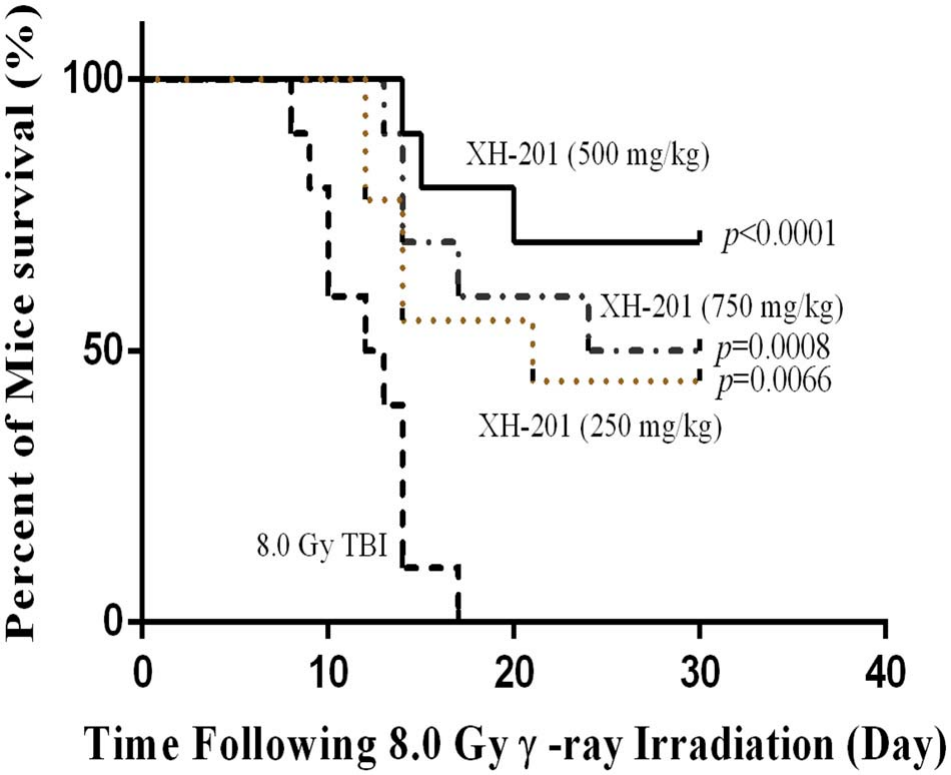
XH-201 administration increased survival after TBI *in vivo*. Mice (*n* = 10) were exposed to 8.0 Gy of ^137^Cs γ-rays and the first dose of XH-201 was administered by injection 30 min before TBI, as illustrated in the diagram, and daily doses of XH-201 were administered by intraperitoneal injection. Control mice were irradiated and received injections of vehicle. The data are expressed as the percentage of surviving mice and were analysed using the log-rank (Mantel–Cox) test. *P* vs TBI with vehicle.

**Fig. 4.**
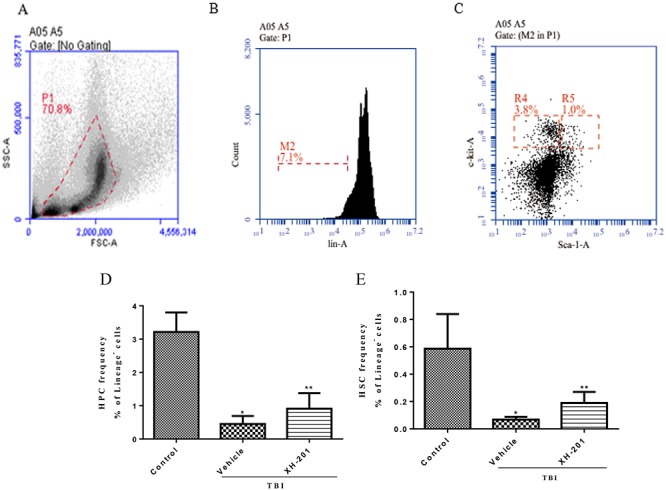
XH-201 ameliorates TBI-induced myelosuppression. (**A**). Representative flow cytometry plots of BMMNCs cells. (**B**) Representative flow cytometry plots of lineage^−^ cells. (**C**) Representative flow cytometry plots of HPCs (Lineage^−^scal^−^c-kit^+^) and HSCs (Lineage^−^scal^+^c-kit^+^). (**D**) The percentage of HPCs among lineage-negative cells. (**E**) The percentage of HSCs among lineage-negative cells. Before the mice were exposed to 4.0 Gy TBI, they were treated with XH-201 (500 mg/kg) as described in the text. Sham-irradiated control mice and XH-201-treated mice were also included. The results were analysed by unpaired *t*-test. ^*^P < 0.01 vs control, ^**^P < 0.05 vs 4.0 Gy with vehicle.

**Fig. 5.**
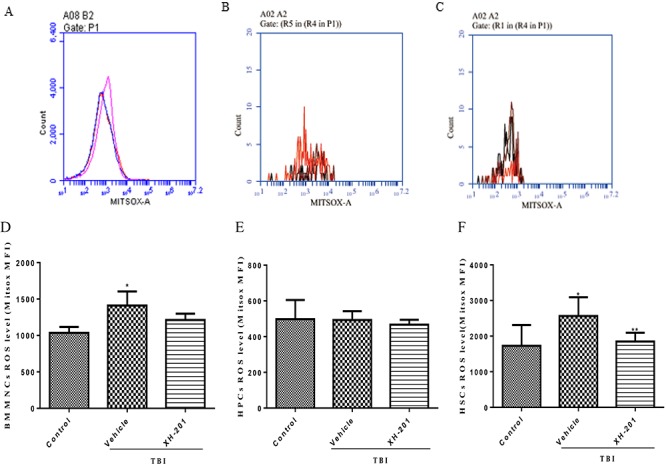
Effects of XH-201 on the levels of ROS in BM hematopoietic cells. Representative analysis of ROS expression in BMMNCs, HPCs and HSCs by flow cytometry. BM hematopoietic cells were isolated from mice 10 days after TBI and then immunostained with antibodies against. Representative ROS flow cytometry graphs are shown for (**A**) BMMNCs, (**B**) HPCs and (**C**) HSCs. ROS levels are shown for (**D**) BMMNCs, (**E**) HPCs and (**F**) HSCs. Before the mice were exposed to 4.0 Gy TBI, they were treated with XH-201 (500 mg/kg) 24 h and 1 h before a sub-lethal dose (4.0 Gy) of TBI, Sham-irradiated control mice and XH-201-treated mice were also included. BMMNCs were collected and detected as described in the Materials and Methods section. The results were analysed by unpaired *t-*test. ^*^P < 0.05 vs control, ^**^P < 0.05 vs 4.0 Gy with vehicle.

**Fig. 6.**
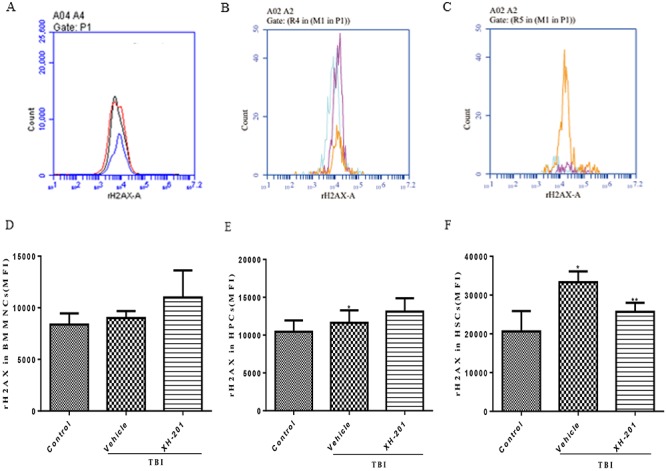
XH-201 regulates TBI-induced γ-H2AX expression in BM hematopoietic cells. Mice were sham-irradiated as a control or irradiated with 4.0 Gy TBI and then treated with XH-201 as described in the text. BM hematopoietic cells were isolated from mice 10 days after TBI and then immunostained with antibodies against. Representative H2AX phosphorylation flow cytometry graphs are shown for (**A**) BMMNCs, (**B**) HPCs and (**C**) HSCs. H2AX phosphorylation is shown for (**D**) BMMNCs, (**E**) HPCs and (**F**) HSCs. Mice were treated with XH-201 (500 mg/kg) 30 min before exposure to 4.0 Gy TBI. BM cells were collected on day 10 and detected as described in the Materials and Methods section. Results were analysed by unpaired *t*-test. ^*^P < 0.01 vs control, ^**^P < 0.05 vs 4.0 Gy with vehicle.

#### Evaluation of the radioprotective activity and cytotoxicity of target compounds

To assess the radioprotective effect of XH-201 and XH-202 on BM cells, we performed luminescence assays to evaluate cell viability. As shown in Fig. 2A–D, compared with the sham-irradiated group, BM cells viabilities decreased significantly after irradiation exposure. However, the viability of irradiated BM cells increased after treatment with XH-201 (10^−5^ and 10^−6^ mol/L, *P* < 0.05) but XH-202 treatment did not show significant radioprotection. These results indicated that XH-201 treatment ameliorates IR-induced injuries in mouse BM cells. Moreover, in order to assess how XH-201 ameliorates TBI-induced BM injury, we measured mitochondrial ROS levels and histone H2AX phosphorylation in BMMNCs after 4.0 Gy radiation. As shown in Fig. 2E and F, compared with the control group, the ROS levels in BMMNCs (*P* < 0.05) increased after 4.0 Gy exposure, while XH-201 treatment reduced the ROS levels (*P*< 0.05). In addition, histone H2AX phosphorylation was higher in BMMNCs irradiated alone compared with the control group, whereas XH-201 treatment decreased the H2AX phosphorylation in BMMNCs (*P* < 0.05). Overall, these data demonstrated that XH-201 can effectively suppress the radiation injury of BMMNCs *in vitro*.

#### XH-201 increased survival after exposure to 8.0 Gy TBI

To assess the protective effects of XH-201 on TBI-induced lethality in mice, we first observed the survival rate of mice for 30 days *in vivo*. As shown in Fig. 3, Kaplan–Meier analysis of survival indicated that the average survival time of the TBI group is 12 days. In contrast, in the irradiated mice treated with 250 mg/kg XH-201, 500 mg/kg XH-201 and 750 mg/kg XH-201, mortalities of the mice at day 30 were 68, 86 and 77%, respectively. The survival rate in the middle-dose XH-201 group was significantly higher than that in the TBI group, suggesting that XH-201 effectively protects mice from irradiation injury.

#### XH-201 attenuates myelosuppression in mice after 4.0 Gy TBI

To examine the protective effect of XH-201, the mice were exposed to 4.0 Gy TBI, which mainly induced damage to the hematopoietic system. As shown in Table 1, the WBC, RBC, HGB, HCT and PLT counts decreased in the TBI group (*P* < 0.05) compared with the respective counts in the control group. Treatment with XH-201 alleviated the TBI-induced damage to the peripheral blood cells and increased the WBC and PLT counts. These results showed that XH-201 can relieve the myelosuppression induced by 4.0 Gy TBI.

**Table 1:** Variation in peripheral blood parameters of mice (*n* = 5)

Group	WBC (×10^3^/mm^3^)	RBC (×10^3^/mm^3^)	HGB (g/dl)	HCT (%)	PLT (×10^3^/mm^3^)
Ctr	10.46 ± 0.69	9.26 ± 0.42	130.8 ± 7.00	31.02 ± 2.05	719.3 ± 37.73
Vehicle+IR	1.31 ± 0.05^*^	7.56 ± 0.03^*^	110.8 ± 1.16^*^	27.76 ± 0.14	391.6 ± 27.81^*^
XH-201 + IR	2.55 ± 0.48^**^	7.35 ± 0.42	106.0 ± 4.53	26.76 ± 1.13	491.4 ± 29.84^**^

Mice were sham-irradiated as a control group (Ctr) or irradiated with 4.0 Gy total body irradiation (TBI). Blood was collected and cells were counted after the mice were euthanized 10 days after receiving 4.0 Gy TBI. The data are expressed as the mean ± SD (*n* = 5 for each group).

^*^ *P* < 0.05 vs control;

^**^ *P* < 0.05 vs 4.0 Gy with vehicle

In addition, the percentages of HPCs (Lineage^−^scal^−^c-kit^+^) and HSCs (Lineage^−^scal^+^c-kit^+^) in BM from control and irradiated mice treated with XH-201 for 10 days after 4.0 Gy TBI were also analysed. TBI caused a decrease in HPCs and HSCs in mice treated with XH-201 compared with the control group. However, treatment of irradiated mice with XH-201 led to a marked recovery of HPCs and HSCs in BM, as shown in Fig. 4 and Supplementary Fig. S11, see online supplementary material. This finding indicated that XH-201 treatment effectively relieved IR-induced BM hematopoietic cell injury.

#### XH-201 inhibits TBI-induced oxidative stress in HSCs

TBI may induce the production of free radicals, contributing to cell and tissue damage [30]. To identify the mechanism underlying the effect of XH-201, we examined whether XH-201 might ameliorate TBI-induced BM suppression via the inhibition of ROS production. As shown in Fig. 5, there were increased ROS levels in BMMNCs (1412 ± 86.39) in vehicle-treated mice after 4.0 Gy TBI compared with those of the control group. But 500 mg/kg XH-201 treatment did not significantly reduce the ROS levels of BMMNCs (1212 ± 38.77) after exposure to 4.0 Gy TBI. Furthermore, ROS levels of HPCs did not show significant differences. However, whereas the ROS levels in HSCs (2566 ± 235.4) were significantly elevated in vehicle-treated mice after TBI compared with non-irradiated HSCs,500 mg/kg XH-201 treatment significantly reduced ROS levels (1850 ± 123.3, *P* < 0.05). Collectively, these data suggest that TBI causes persistent oxidative stress in HSCs, whereas XH-201 significantly attenuates the increased levels of ROS, thus implying that XH-201 may scavenge free radicals in the HSCs to alleviate TBI-induced damage.

#### XH-201 inhibits TBI-induced increases in DNA double-strand breaks in HSCs

To determine whether XH-201 treatment could reduce TBI-induced DNA damage, we examined histone H2AX phosphorylation expression in BMMNCs, HPCs and HSCs by flow cytometry analysis, which has been widely used as a marker for DNA double-strand breaks (DSBs). DSBs result in the phosphorylation of H2AX histones around each DSB to form γH2AX foci and can be detected by a Ser139-specific antibody. H2AX phosphorylation has been used for quantifying DSBs in many studies [31, 32]. As shown in Fig. 6, histone H2AX phosphorylation was higher in BMMNCs (9005 ± 301.7) and HPCs (11624 ± 735) in vehicle-treated mice irradiated alone compared with the un-irradiated control group; but there is no significant difference of H2AX phosphorylation in BMMNCs (10990 ± 1174) and HPCs (13108 ± 790.3) of mice with XH-201 + radiation. However there was an increase in H2AX phosphorylation in HSCs (33331 ± 1391) of the IR group compared with the un-irradiated control group (20620 ± 2353). Treatment with 500 mg/kg XH-201 decreased H2AX phosphorylation in HSCs (25676 ± 1345, *P* < 0.05) compared with the irradiated mice.

## DISCUSSION

Among the various tissues and organs of victims that receive moderate or high doses of TBI, BM is one of the most radiosensitive tissues in the body. Exposure to moderate doses of TBI causes acute and transient BM inhibition, which primarily impairs HPCs and to a lesser extent HSCs [33,34].

A structure–activity relationship (SAR) study revealed that Cl, F and OCF3 substitution in the aryl ring plays a key role in radioprotective efficiency [11]. The introduction of halogen groups at the 4-position of the benzene ring enhanced the radioprotective effect [12]. For XH-201, Cl is at the *ortho* position of the pyridine ring and Cl substitution of XH-202 is at the *meta* position. XH-201 showed higher free radical scavenging ability in the DPPH experiments. The SAR between XH-201 and XH-202 needs to be verified by more study results because their mechanism of radioprotection is complex. We will continue to explore and carry out further research in this area. In this study, we examined whether XH-201 might mitigate IR-induced BM injury. Ex-Rad showed a significant protective effect against IR when it was administered by subcutaneous injection or intragastric administration [10, 35]. Our findings showed that administration of 500 mg/kg XH-201 not only protected mice from IR-induced lethality but also mitigated TBI-induced hematopoietic injury. Treatmet with 500 mg/kg XH-201 ameliorated the survival rates of mice after exposure to a lethal dose of radiation, confirming that XH-201 provided protection against radiation injury. One of the most important side-effects of radiation syndrome is hematopoietic damage [36, 37]. Therefore, we used an established mouse model to study the effects of XH-201 on the hematopoietic system [38, 39]. Radioprotection by Ex-Rad was associated with significant protection of leukocytes and platelets in peripheral blood in BM [40]. We found that XH-201 improved the numbers of BMMNCs and PLTs and increased the percentage of HPCs and HSCs, which suggested that XH-201 not only protected the cells against IR injury but also improved their function. Research shows that the XH-201 analogue ON 01210.Na was able to accelerate the post-exposure hematopoietic recovery process, as evidenced by a greater increase in peripheral WBC and platelet counts, higher BM cellularity and higher numbers of GM-CFUs in the drug-treated groups than in the vehicle groups [41].

It is well known that in radiation tissue, oxidative stress caused by elevated ROS levels contributes to tissue damage. In this study, we found that oxidative stress was ameliorated by treatment with XH-201. DNA damage and apoptosis are caused by oxidative stress resulting from excessive ROS production. IR induces DNA to produce base damage and changes, cross-linking, single-strand breaks, DSBs and several other different types of damage. Among them, DSB is the most harmful DNA damage to cells caused by unrepaired or misrepaired DNA, which eventually leads to chromosome breakage and translocation, resulting in human-related diseases including cancer [25]. In many studies, H2AX phosphorylation is used to quantify DSBs [42]. Previous studies have indicated that the inhibition of ROS reduced IR-induced expression of γ-H2AX [43]. In addition, IR-induced hematopoietic toxicity was alleviated by Ex-Rad through mitigating the ATM-p53-mediated DNA damage response [30]. Moreover, a mechanistic study demonstrated that its radioprotective effects may include the prevention of p53-dependent apoptosis [29]. In this study, we found that XH-201 reduced the increases in γ-H2AX expression in HSCs after TBI, which verified the effects of XH-201 on DNA damage.

## CONCLUSION

In the present study, we designed and prepared two new aryl sulfone derivatives XH-201and XH-202 and evaluated their radioprotective efficacies. Our studies illustrated the effectiveness of XH-201 for protection against radiation-induced injury in mice and indicated that XH-201 might potentially be used as an efficacious medical radiation countermeasure. We indicated that XH-201 had a protective effect on TBI-induced myelosuppression, and we have also found that XH-201 has the ability to rescue DNA damage. More research is needed to determine whether XH-201 mediates the role of ROS-related pathways in its radiation protection, or whether there are significant changes to the expression of other genes and proteins that contribute to XH-201 protection.

## Acronyms

ROS Reactive oxygen species

TLC Thin layer chromatography

NMR Nuclear magnetic resonance

HR-MS High resolution mass spectrometry

HPLC High performance liquid chromatography

ESI Electrospray ionization

DMSO Dimethyl sulfoxide

HoAc Acetic acid

EtOH Ethanol

PerCP Peridinin-ChloroplyII-Protein Complex

ANOVA Analysis of Variance

LSD Least-Significant Difference

SEM Standard Error of Mean

GM-CFUs Granulocyte/Macrophage Colony-Forming Units

## FUNDING

This work was supported by the CAMS Innovation Fund for Medical Science (2017-I2M-3-019) from the Chinese Academy of Medical Sciences & Peking Union Medical College, Key Scientific and Technological Support Projects of Tianjin Key R&D Program (19YFZCSY00350) and Tianjin Major Scientific and Technological Projects of New Drug Creation (17ZXXYSY00090). This work was also supported by a Project Funded by China Postdoctoral Science Foundation (2019 M650565) and the National Natural Science Foundation of China (No. 81972975).

## CONFLICT OF INTEREST

None declared.

## Supplementary Material

Supporting_Information_for_XH-201_rraa009
