# Supplementary material for: Protective effects of new aryl sulfone derivatives against radiation-induced hematopoietic injury
Source: J Radiat Res. 2020 Mar 16;61(3):388–98. doi: 10.1093/jrr/rraa009 (PMC7299261; doi:10.1093/jrr/rraa009)
Supplement: Supporting_Information_for_XH-201_rraa009 [file supporting_information_for_xh-201_rraa009.docx]

**Supporting Information**

**Protective Effects of New Aryl Sulfone Derivative against Radiation‐Induced Hematopoietic Injury**

**Characterization of XH-202**

White solid; ^1^H NMR (400 MHz, D_2_O) δ 8.50 (s, 1H), 7.88 (d, *J* = 7.7 Hz, 3H), 7.60 (d, *J* = 7.8 Hz, 2H), 7.53 (d, *J* = 8.3 Hz, 1H), 7.42 (d, *J* = 15.5 Hz, 1H), 7.17 (d, *J* = 15.5 Hz, 1H), 4.74 (d, *J* = 6.1 Hz, 2H). ^13^C NMR (101 MHz, D_2_O) δ 174.57, 148.26, 146.35, 145.79, 141.00, 139.19, 137.95, 133.91, 132.70, 132.04, 129.34, 128.70, 127.50, 124.02, 123.73. ESI-MS: m/z = 360 (M+H) ^+^; HRMS (ESI): m/z [M+H] ^+^calcd for C_15_H_12_ClNNaO_4_S: 360.0073, found: 360.0041.

**NMR spectra for selected compounds**


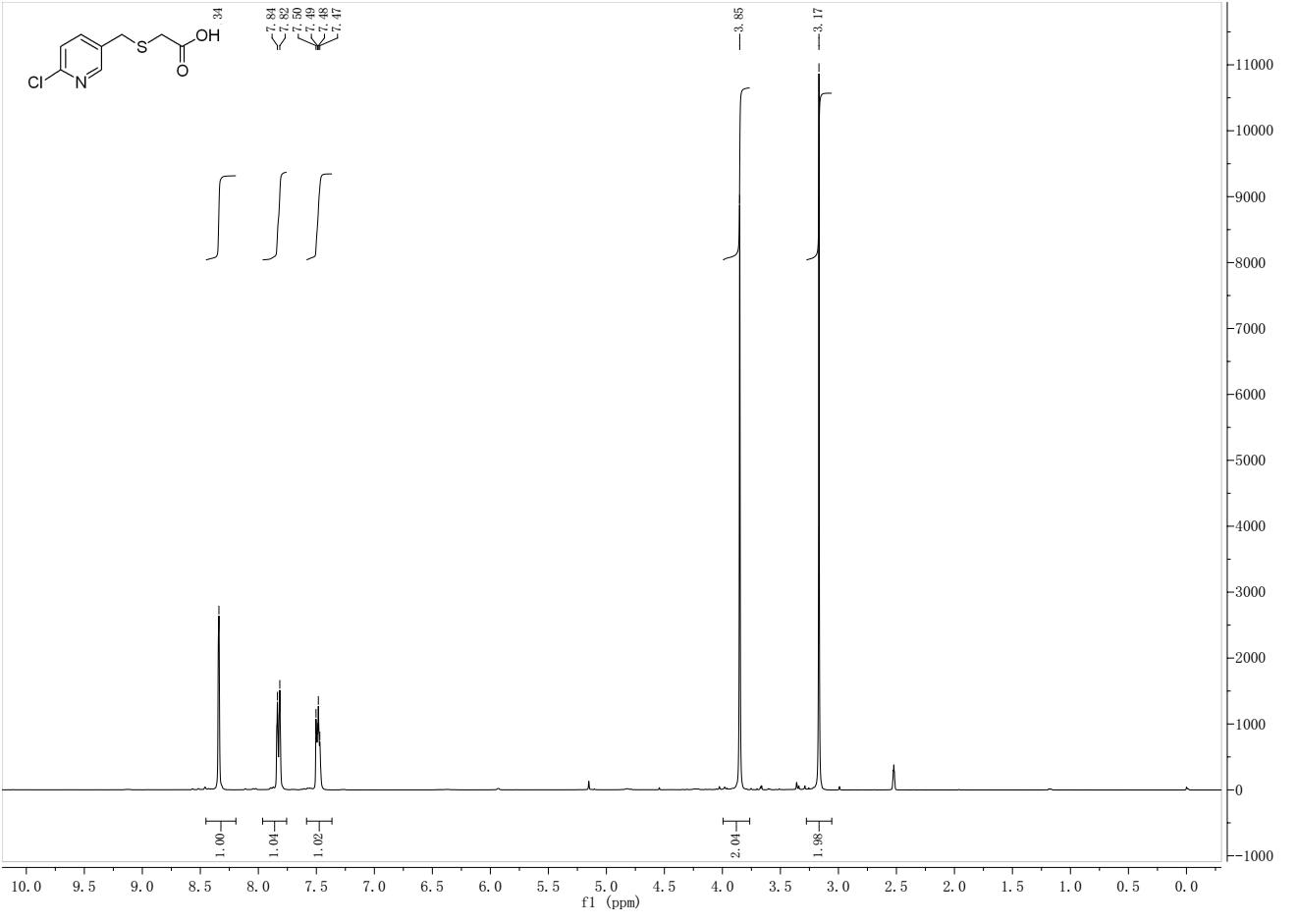


**Figure S1.** ^1^H NMR (400 MHz, DMSO-d6) spectrum of **compound 2a**


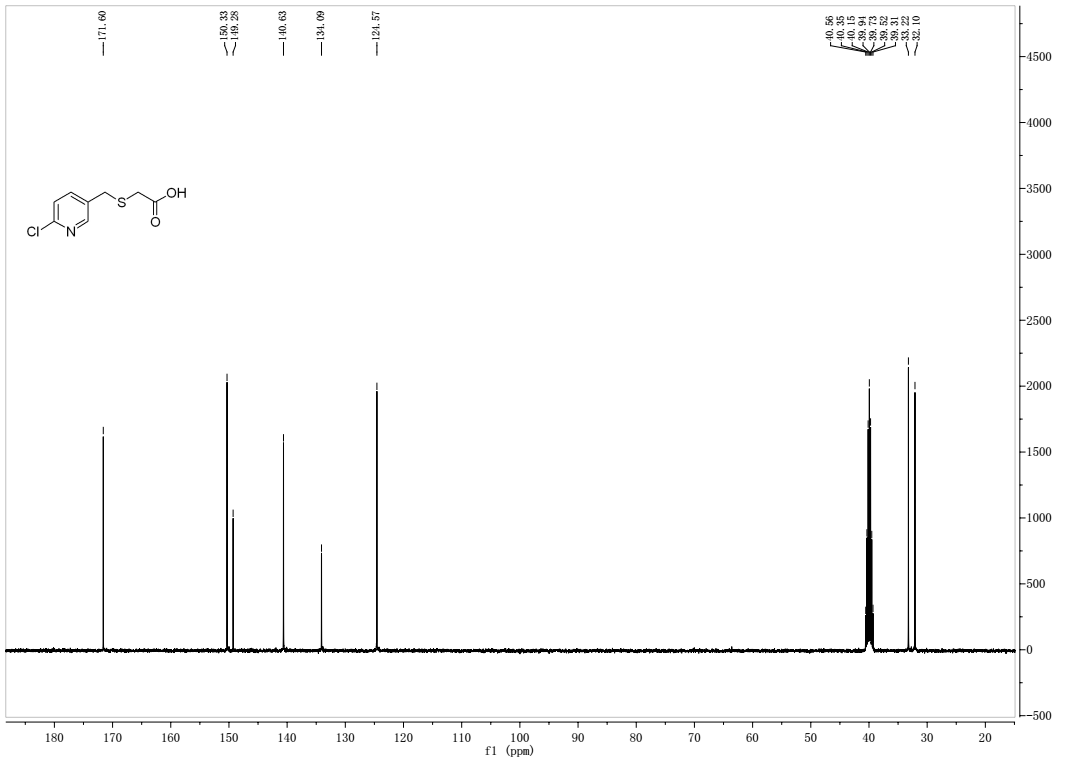


**Figure S2.** ^13^C NMR (101 MHz, DMSO-d6) spectrum of **compound 2a**


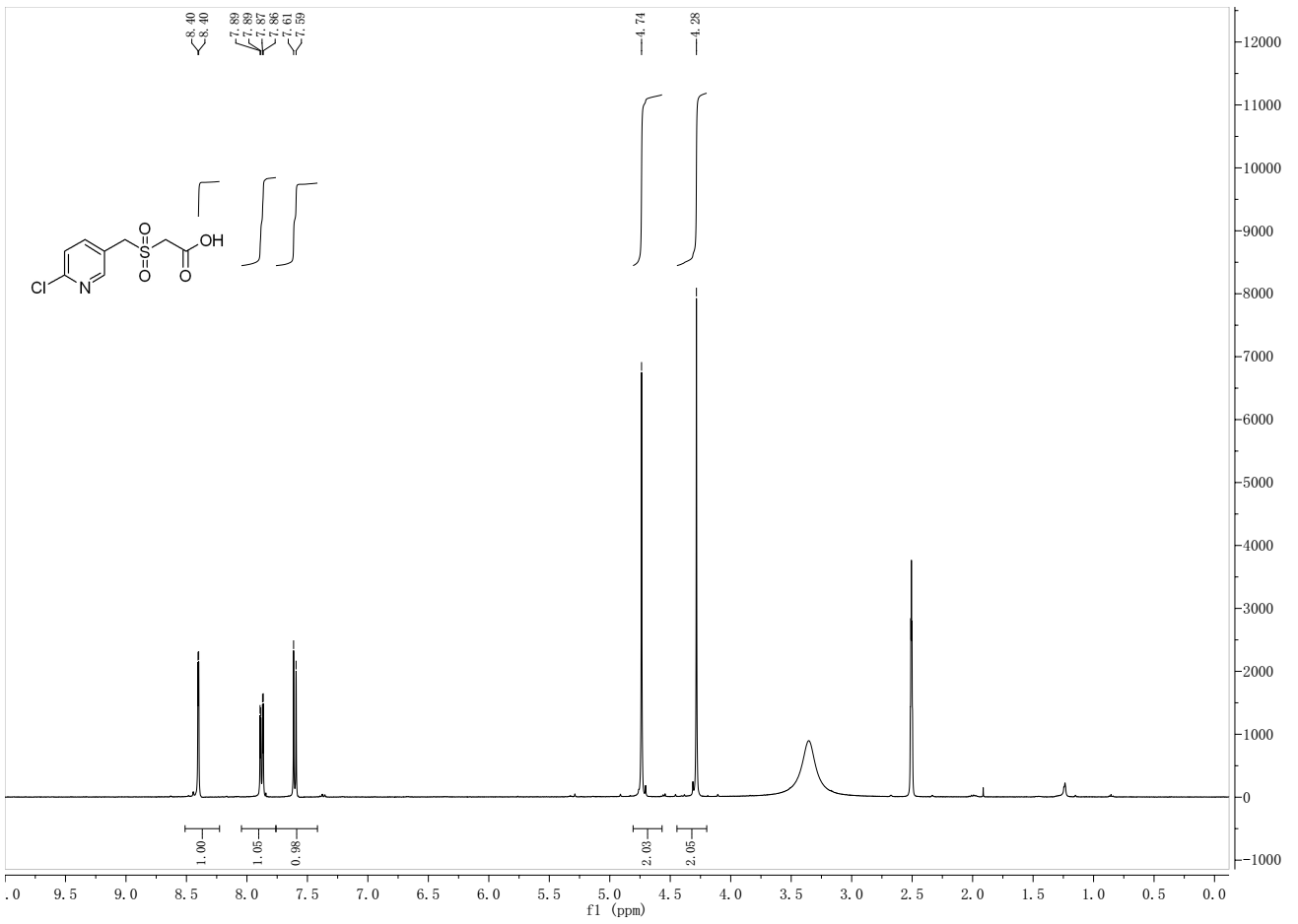


**Figure S3.** ^1^H NMR (400 MHz, DMSO-d6) spectrum of **compound 3a**


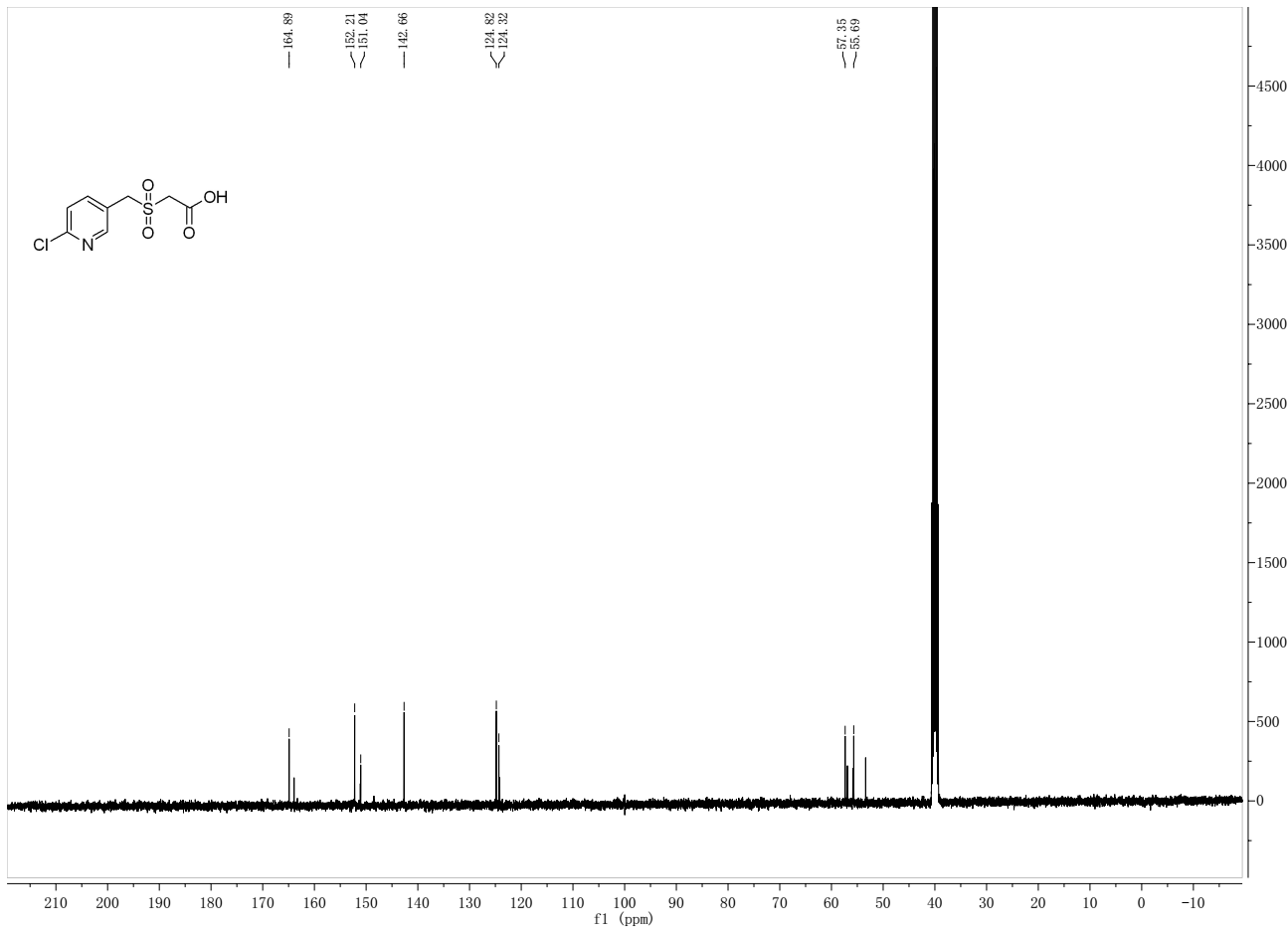


**Figure S4.** ^13^C NMR (101 MHz, DMSO-d6) spectrum of **compound 3a**


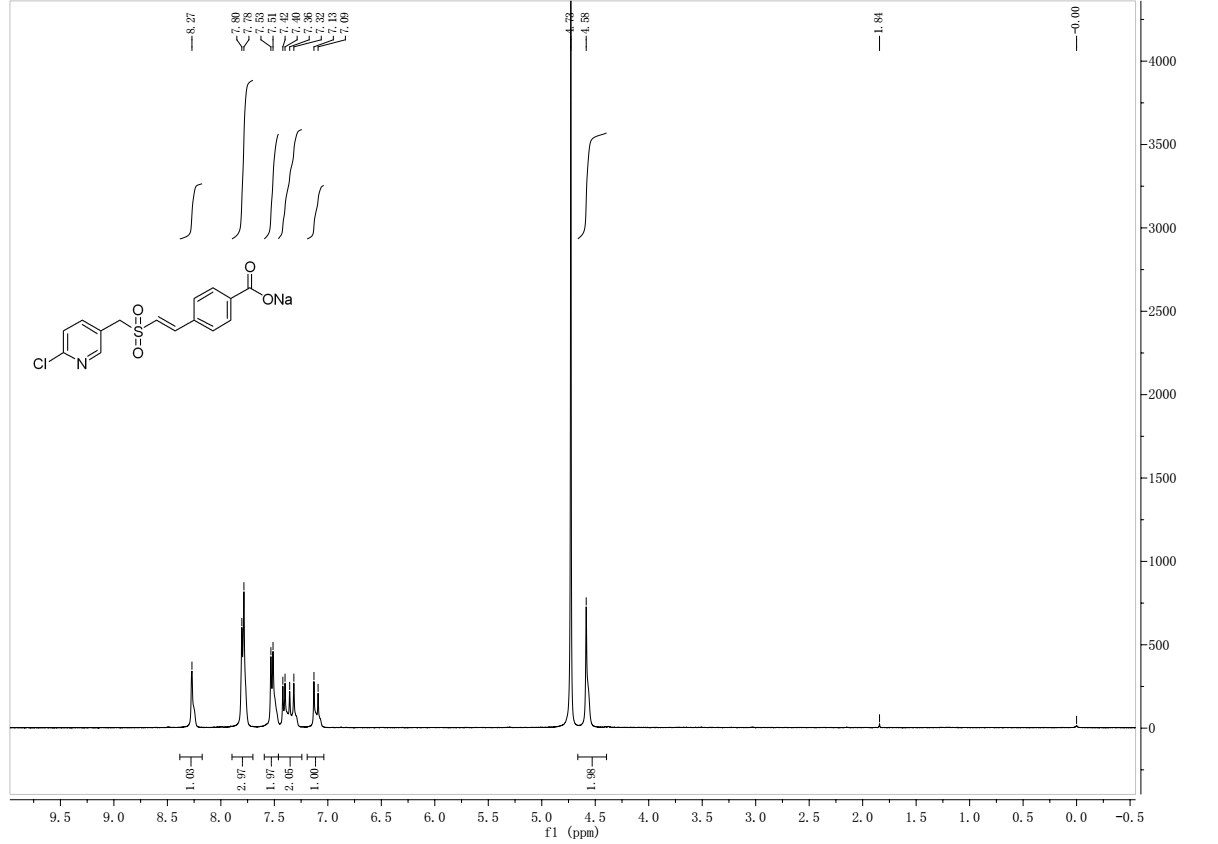


**Figure S5.** ^1^H NMR (400 MHz, D_2_O) spectrum of **XH-201**

**
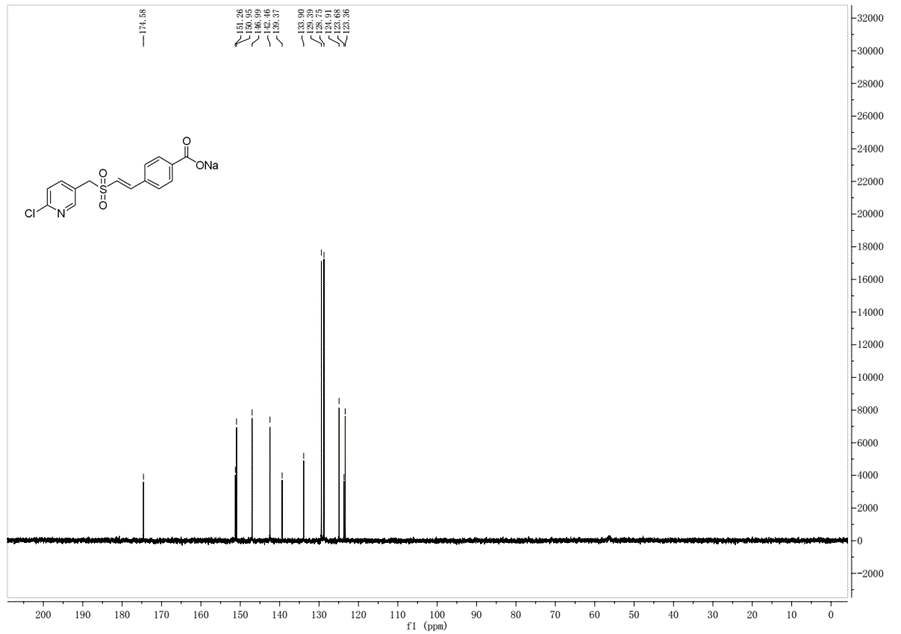
**

**Figure S6.** ^13^C NMR (101 MHz, D_2_O) spectrum of **XH-201**


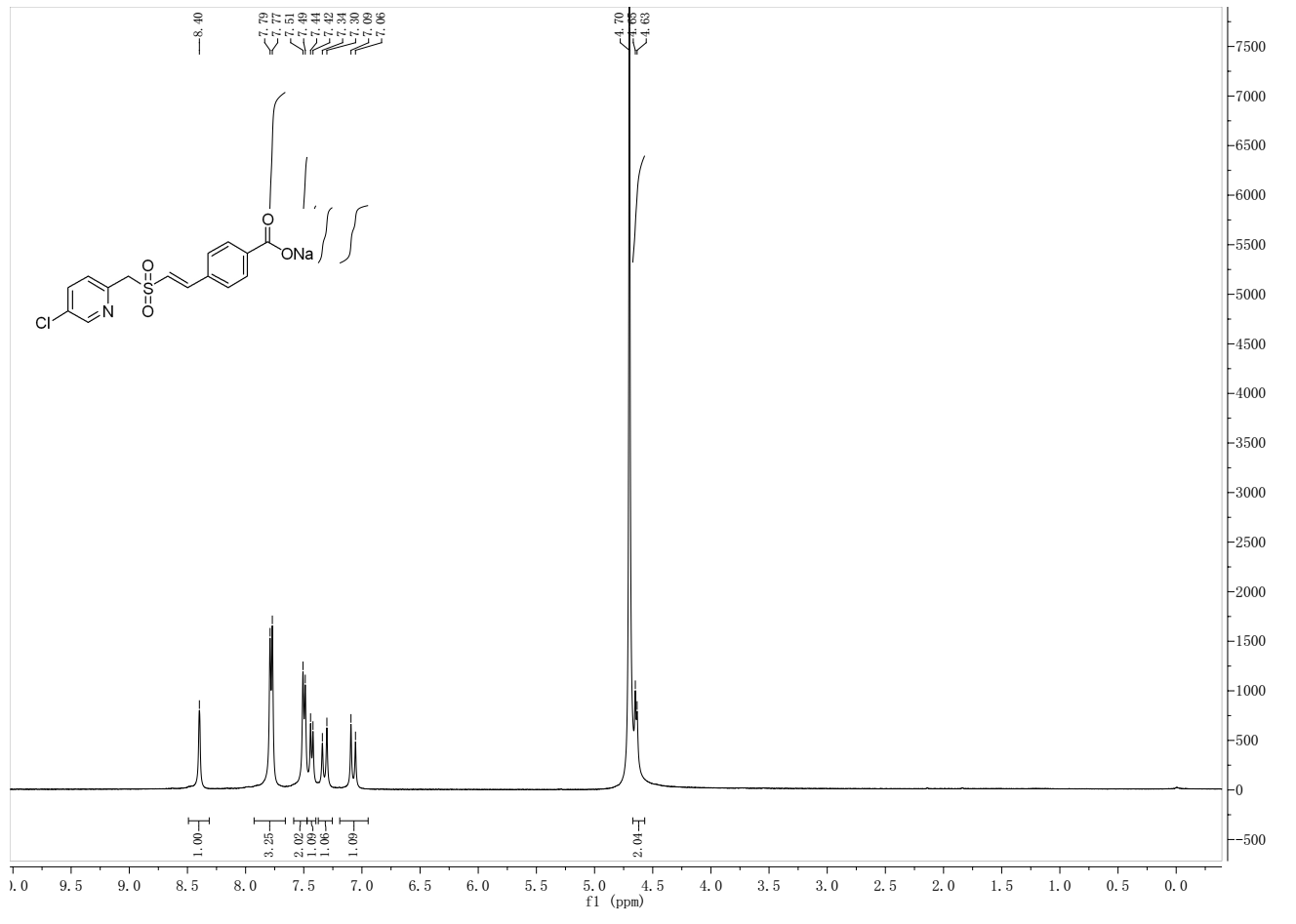


**Figure S7.** ^1^H NMR (400 MHz, D_2_O) spectrum of **XH-202**


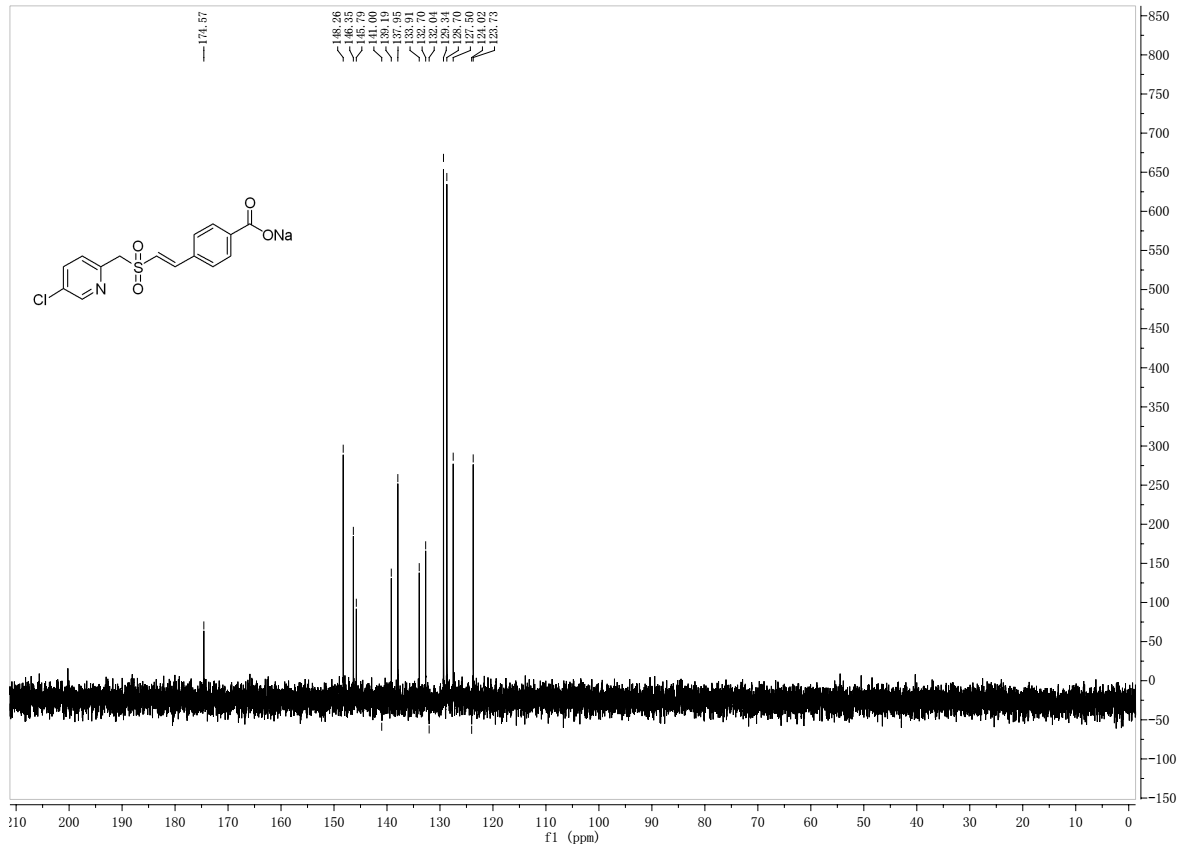


**Figure S8.** ^13^C NMR (101 MHz, D_2_O) spectrum of **XH-202**


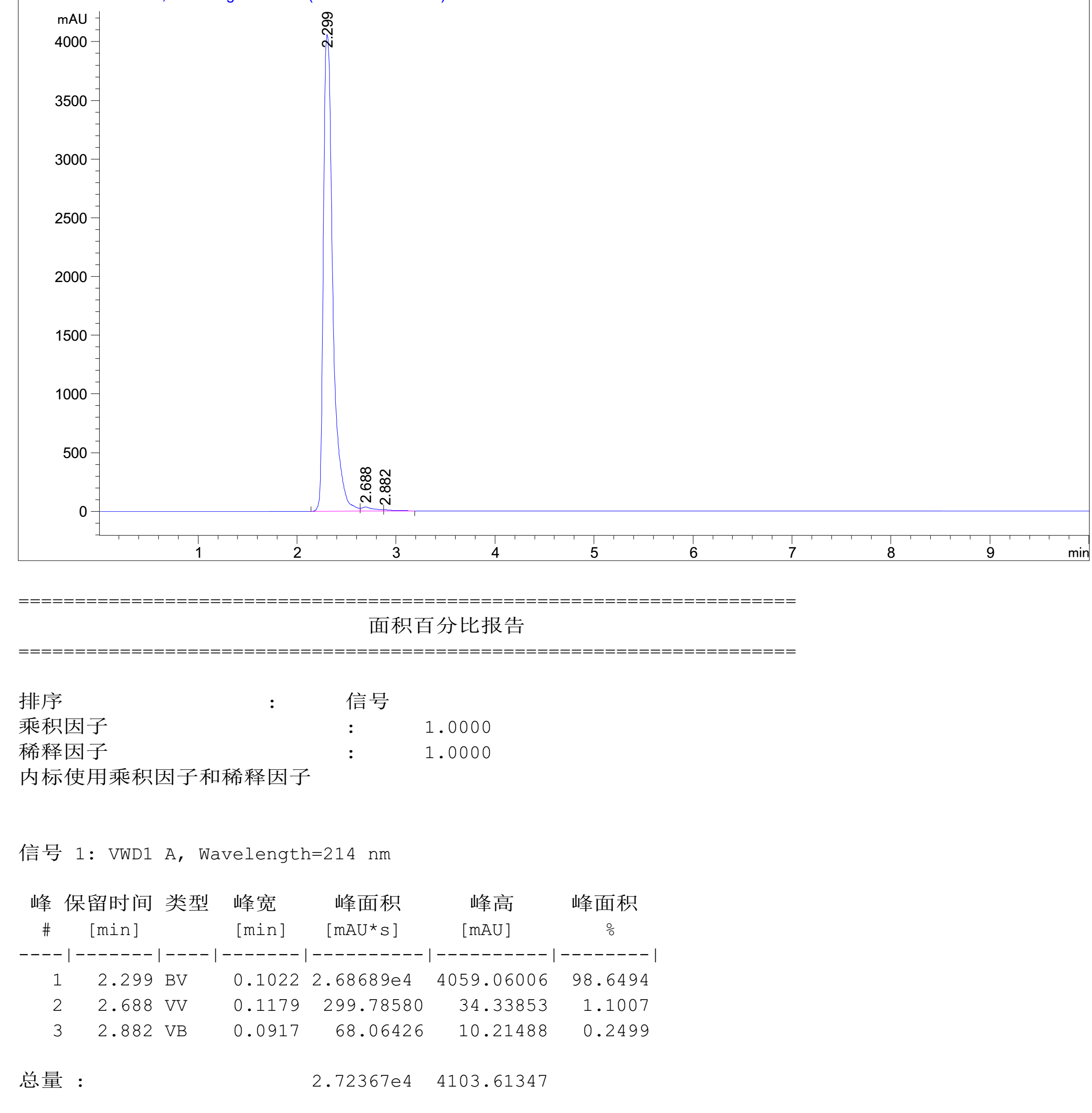


Wavelength = 214 nm

| Peak | RT (min) | Width  (min) | Area (mAU*s) | Height  (mAU) | Area (%) |
| --- | --- | --- | --- | --- | --- |
| 1 | 2.299 | 0.1022 | 2.68689e4 | 4059.06006 | 98.6494 |
| 2 | 2.688 | 0.1179 | 299.78580 | 34.33853 | 1.1007 |
| 3 | 2.882 | 0.0917 | 68.06426 | 10.21488 | 0.2499 |

**Figure S9.** HPLC spectra of **XH-201**


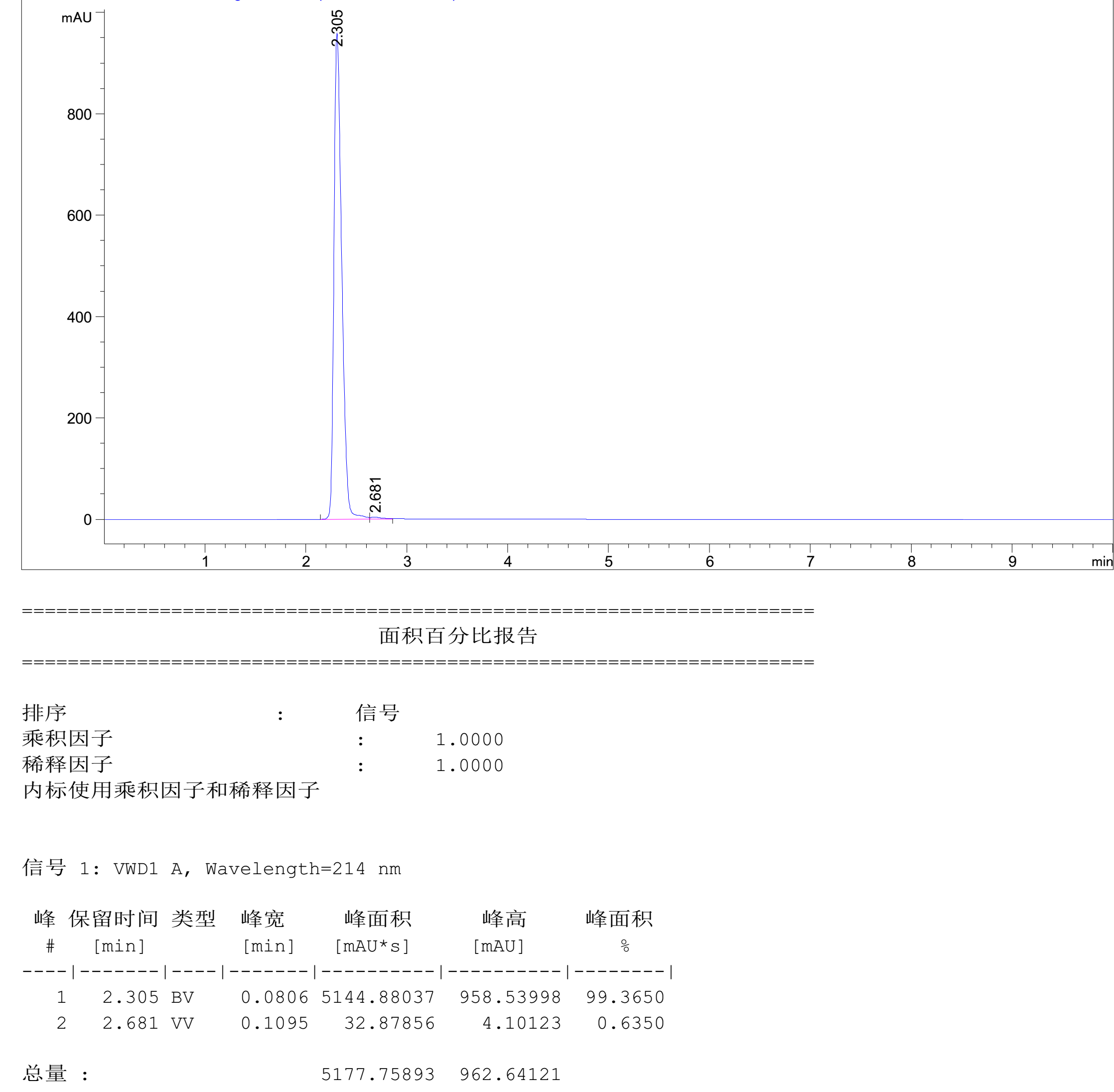


Wavelength = 214 nm

| Peak | RT (min) | Width  (min) | Area (mAU*s) | Height  (mAU) | Area (%) |
| --- | --- | --- | --- | --- | --- |
| 1 | 2.305 | 0.0806 | 5144.88037 | 958.53998 | 99.3650 |
| 2 | 2.681 | 0.1095 | 32.87856 | 4.10123 | 0.6350 |

**Figure S10**. HPLC spectra of **XH-202**


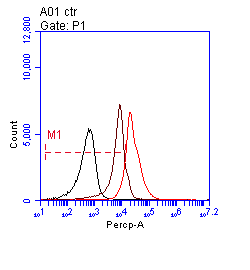


**Figure S11.** The graph of bone marrow cell in FL3 channel（Percp）. The black line is the bone marrow cells with no staining, the purple line is the cells with no staining with mixed lineage antibodies, which were  stained with only  secondary  streptavidin-Percp, the red line is the bone marrow cells which were stained with  secondary streptavidin-Percp after staining with biotin-conjugated antibodies specific for murine CD5, CD11b, CD45R/B220, Ter-119, and Gr-1.
